# Benzophenone guttiferone A from *Garcinia achachairu* Rusby (Clusiaceae) Presents Genotoxic Effects in Different Cells of Mice

**DOI:** 10.1371/journal.pone.0076485

**Published:** 2013-11-08

**Authors:** Peterson Menezes Terrazas, Eduardo de Souza Marques, Luisa Nathália Bolda Mariano, Valdir Cechinel-Filho, Rivaldo Niero, Sergio Faloni Andrade, Edson Luis Maistro

**Affiliations:** 1 Universidade Estadual Paulista – UNESP – Faculdade de Filosofia e Ciências, Departamento de Fonoaudiologia, Marília, SP, Brazil; 2 Programa de Pós-Graduação em Ciências Farmacêuticas, Núcleo de Investigações Químico-Farmacêuticas, Universidade do Vale do Itajaí, UNIVALI, Itajaí, Santa Catarina, Brazil; National Research Council, Italy

## Abstract

Benzophenones from natural sources and those of synthetic analogues present several reports of potent biological properties, and Guttiferone A represents a promising medicinal natural compound with analgesic and gastroprotective profiles. Considering that there are no reports that assess the genetic toxicity of Guttiferone A, the present study was undertaken to investigate the genotoxic potential of this benzophenone isolated from seeds of *Garcinia achachairu* in terms of DNA damage in different cells of Swiss albino mice using the comet assay, and its clastogenic/aneugenic effects in bone marrow cells *in vivo* by the micronucleus test. Cytotoxicity was assessed by scoring polychromatic (PCE) and normochromatic (NCE) erythrocytes ratio. Guttiferone A was administered by oral gavage at doses of 15, 30 and 60 mg/kg. The results showed that Guttiferone A produced genotoxic effects in leukocytes, liver, bone marrow, brain and testicle cells and clastogenic/aneugenic effects in bone marrow erythrocytes of mice. The PCE/NCE ratio indicated no cytotoxicity. Since guttiferone A is harmful to the genetic material we suggest caution in its use by humans.

## Introduction

 The genotoxic assessment of plants and its compounds with possible therapeutic properties is very important because DNA damage induced by mutagens can play a key role in the process of carcinogenesis and inherited genetic diseases.

 Plants belonging to the Clusiaceae (or Guttiferae) family are distributed mainly in tropical regions. This family comprises about 40 genera and 1,200 species, the genus *Garcinia* (ex-*Rheedia*) being the most numerous, with about 400 species widely distributed in tropical Brazil, Polynesia, New Caledonia, Africa, and Asia [1]. *Garcinia achachairu* Rusby belongs to the genus *Garcinia*; which is widely distributed in the region of Santa Cruz, Bolivia and is well-adapted in Brazil, where it is easy to cultivate and harvest. This plant is used in Bolivian folk medicine for its healing, digestive, and laxative properties [2]. In Brazil, it is popularly known as “achachairu” and is used in folk medicine to treat rheumatism, inflammation, pain and gastric disorders [3,4]. 

 Phytochemical characterization of seed extract of *G. achachairu* reveals the presence of benzophenones, xanthones and bioflavonoids, such as guttiferone N, garcinol, isogarcinol, guttiferone M, camboginol, xanthochymol and guttiferone A, with benzophenone guttiferone A as the major compound [5,6]. Benzophenones are known to exhibit various biological activities, such as cytotoxic, antimicrobial, antiviral and antioxidant activities [7]. Niero et al. [8] reported that extracts obtained from *G. achachairu*, and its major compound guttiferone A, produce gastroprotective effects against induced gastric lesions in mice. The same research group reported that the seed extract of *G. achachairu* and the compound guttiferone A present antinociceptive effects [5].

 Although benzophenones from natural sources and those of synthetic analogues present several reports of potent biological properties, and guttiferone A represents a promising medicinal natural compound with analgesic and gastroprotective profiles, to the best of our knowledge, there are no reports that assess its genetic toxicity. Therefore, the present study was undertaken to investigate the genotoxic effects of the benzophenone guttiferone A in terms of DNA damage in peripheral blood, liver, bone marrow, brain and testicle cells of mice, and its clastogenic/aneugenic potential in bone marrow cells *in vivo*.

## Materials and Methods

### Plant material

*Garcinia achachairu* is not a protected species in Brazil, therefore, no specific permissions were required to its use in this research. Due to fact that fruits of *G. achachairu* are produced for commercialization, the collect of the different plant's parts (leaves, seeds and branches) was undertaken in commercial plantation in the town of Camboriú, SC, Brazil in March 2007, with permission of the owner of the land. Samples were identified by Dr. Oscar B. Iza (University of Itajaí Valley). A voucher specimen was deposited at the Barbosa Rodrigues Herbarium (Itajaí, SC, Brazil) under number HBR 52637

### Extraction and isolation

**Figure 1:**
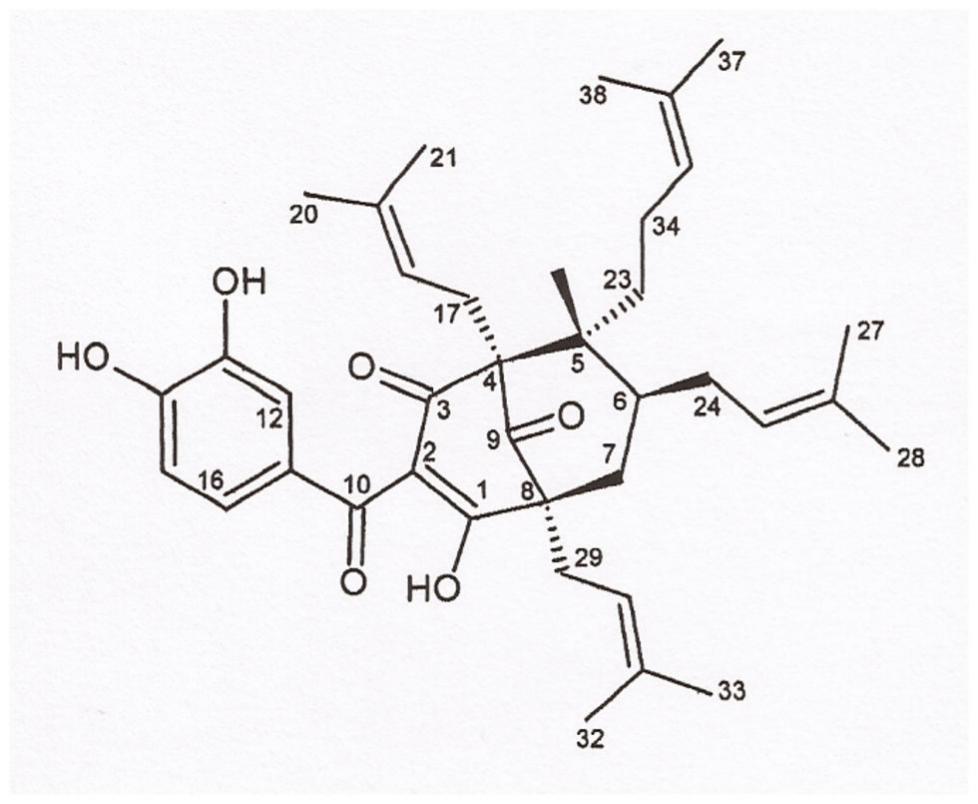
Molecular structure of guttiferone A isolated from *G. achachairu*.

Seeds (250g) of *G. achachairu*, air-dried and powdered, were extracted at room temperature with methanol (2x 1000mL) for seven days. The macerated seeds were filtered and concentrated under reduced pressure, yielding 9.01g (3.6%) of crude methanol seed extract. In view of the higher biological activity exhibited by the methanolic seed extract, this was chromatographed (5.0g) on a silica-gel column (0.063–0.20 mm, 84.0g, 2.5 x 50 cm, Merck, Darmstadt, Germany) and eluted with a solution of CHCl_3_:MeOH (starting with 100% of CHCl_3_ and ending with 100% of MeOH) yielding 240 fractions. Those fractions that behaved similarly in thin layer chromatography (TLC) were combined, yielding 660 mg of a yellow solid. The compound was identified as guttiferone A (Figure 1) by TLC and nuclear magnetic resonance (NMR) spectral data in comparison with authentic samples and the literature data [9]. It's purity was estimated at 97.53% by HPLC analysis.

### Reagents

 The agent doxorubicin (DXR, Oncodox®, Meizler) was used as the DNA damaging agent in the comet assay and micronucleus test using Swiss mice. The other main chemicals were obtained from the following suppliers: normal melting point (NMP) agarose (Cat. No. 15510-019 - Invitrogen), low melting point (LMP) agarose (Cat. No. 15517-014 - Invitrogen), sodium salt *N*-lauroyl sarcosinate (L-5125 - Sigma) and ethylenediaminetetraacetic acid (EDTA - Merck).

### Animals and dosing

 The experiments were carried out using 12-week-old male Swiss albino mice (*Mus musculus*), weighing 25-30 g. The animals were acquired from the Universidade Estadual Paulista (UNESP), Botucatu, São Paulo State, Brazil, and housed in polyethylene boxes in a climate-controlled environment (25 ± 4°C, 55 ± 5% humidity) with a 12-h light/dark cycle (7:00 am to 7:00 pm). Food (Nuvilab CR1, Nuvital) and water were available *ad libitum*. The mice were divided into 5 experimental groups of 6 animals each. Guttiferone A was dissolved in 1% Tween 80 aqueous solution and administered in a single dose of 0.3 mL by gavage at concentrations of 15, 30 and 60 mg/kg body weight, chosen on the basis of its gastroprotective effects at 30 mg/Kg [8]. The negative control group received 1% Tween 80 aqueous solution by gavage, and the positive control group received an intraperitoneal injection of doxorubicin (DXR) at 80 mg/kg body weight. The animals used in this study were sacrificed by cervical dislocation after anesthesia (chloral hydrate 10%, 4 mL/kg b.w., i.p.). The Animal Bioethics Committee of the Faculdade de Medicina de Marília (CEP/FAMEMA, Marília, São Paulo state, Brazil) approved the present study on 30 November 2012 (protocol number 1669/12), in accordance with the federal government legislation on animal care.

### Comet Assay

The comet assay (SCGE) was carried out by the method described by Speit and Hartmann [10] and reviewed by Burlinson et al. [11]. Peripheral blood samples from the tail vein were obtained from six Swiss mice of each group, at 4 and 24 h after treatment and before euthanasia. After sacrificing the animals, liver, bone marrow, brain and testicle cell samples were washed in saline solution, in an ice bath. A small portion of liver, brain and testicle (about 4 millimeters in diameter) was transferred to a Petri dish containing 1mL of Hank’s solution (pH 7.5) and then homogenized gently with a small pair of tweezers and a syringe to remove any clumps of cells. An aliquot of 20 µL was removed from the supernatant of each cell type to determine cell viability. Cell counting was performed using a hemocytometer. Cell viability was determined by trypan blue dye exclusion. The number of trypan blue-negative cells was considered as the number of viable cells, and was greater than 90%. Another equal aliquot of cells from each animal was mixed with 120 µL of 0.5% low melting point agarose at 37°C, and rapidly spread onto two microscope slides per animal, pre-coated with 1.5% normal melting point agarose. The slides were coverslipped and allowed to gel at 4°C for 20 min. The coverslips were gently removed and the slides were then immersed in cold, freshly-prepared lysing solution consisting of 89 mL of a stock solution (2.5 M NaCl, 100 mM EDTA, 10 mM Tris, pH set to 10.0 with ~8 g solid NaOH, 890 mL of distilled water and 1% sodium lauryl sarcosine), plus 1 mL of Triton X-100 (Merck) and 10 mL of dimethyl sulfoxide (Merck). The slides, which were protected from light, were allowed to stand at 4°C for 1 h and then placed in the gel box, positioned at the anode end, and left in a high pH (>13) electrophoresis buffer (300 mM NaOH-1 mM EDTA, prepared from a stock solution of 10 N NaOH and 200 mM, pH 10.0, EDTA) at 4°C for 20 min prior to electrophoresis, to allow DNA unwinding. The electrophoresis run was carried out in an ice bath (4°C) for 20 min at 300 mA and 25 V (0.722 V cm^-1^). The slides were then submerged in a neutralization buffer (0.4 M Tris-HCl, pH 7.5) for 15 min, dried at room temperature and fixed in 100% ethanol for 10 min. The slides were dried and stored overnight or longer, before staining. For the staining process, the slides were briefly rinsed in distilled water, covered with 30 µL of 1x ethidium bromide staining solution prepared from a 10x stock (200µg/ml) and coverslipped. The material was evaluated immediately at 400x magnification, using a fluorescence microscope (Olympus BX 50) with a 515-560 nm excitation filter and a 590 nm barrier filter. Only individual nucleoids were scored.

 The extent and distribution of DNA damage indicated by the SCGE assay was evaluated by examining at least 100 randomly selected and non-overlapping cells (50 cells per coded slide) per animal in a blind analysis (six mice per group). These cells were scored visually, according to tail size, into the following four classes: class 0- no tail; class 1- tail shorter than the diameter of the head (nucleus); class 2- tail length 1 to 2 times the diameter of the head; and class 3- tail length more than twice the diameter of the head. Comets with no heads, with nearly all of the DNA in the tail, or with a very wide tail were excluded from the evaluation because they probably represented dead cells [12]. The total score for 100 comets, which ranged from 0 (all undamaged) to 300 (all maximally damaged), was obtained by multiplying the number of cells in each class by the damage class.

### *In vivo* Micronucleus test

The assay was carried out following standard protocols, as recommended by Schmid [13] and Krishna and Hayashi [14]. The same six male mice per group as those used in the comet assay were also used to this protocol. The bone marrow from one femur was flushed out using 2 mL of saline (0.9% NaCl) and centrifuged for 7 min. The supernatant was discarded and smears were made on slides. The slides were coded for a “blind” analysis, fixed with methanol and stained with Giemsa. For the analysis of the micronucleated cells, two thousand polychromatic erythrocytes (PCE) per animal were scored to determine the clastogenic/aneugenic property of the extract. To detect possible cytotoxic effects, the PCE-NCE (normochromatic erythrocytes) ratio of 200 erythrocytes/animal was calculated [15]. The cells were blindly scored using a light microscope at 1000x magnification. The mean number of micronucleated polychromatic erythrocytes (MNPCE) in individual mice was used as the experimental unit, with variability (standard deviation) based on differences among animals within the same group.

### Statistical analysis

 After verifying normal distribution (KS normality test), the data obtained from the comet assay and micronucleus test were submitted to analysis of variance (ANOVA) and the Tukey-Kramer multiple comparison test, using the GraphPad Instat® software (version 3.01). The results were considered statistically significant at *P* < 0.05.

## Results

Table 1 shows the DNA damage in peripheral blood cells, collected 4 and 24 h after the treatment, and liver, bone marrow, brain and testicle cells collected 24 h after guttiferone A treatment, as detected by the single cell gel (comet) assay. Trypan blue staining showed that the cell viability for all the cells was greater than 90 %, confirming the absence of cytotoxicity observed by the PCE-NCE ratio in the micronucleus (MN) test (Table 2), for the three tested doses of the test compound. No deaths, morbidity or distinctive clinical signs were observed in the treated animals following guttiferone A treatment. As expected, when the positive control was compared with the negative control group, we found that DXR induced a significant increase (P < 0.001 or greater) in comet assay DNA migration for all the cell types analyzed, indicating the validity of the species selected, and of the study design, to detect genotoxic effects. In all the analyzed cell types, significant increases in DNA damage (P < 0.05) were found between the negative control group and experimental groups treated with 15, 30 and 60 mg/Kg doses of guttiferone A. In the peripheral blood samples only, the lesser dose of the test compound did not produce a significant increase in DNA damage. The DNA damage observed did not show a direct dose-response of guttiferone A for the majority of the cell types studied. In the cells with significant DNA damage, most of the damage was minor (class 1), with only a few cells showing a large amount of damage (classes 2 and 3).

**Table 1:** DNA migration in the comet assay for assessing the genotoxicity of Guttiferone A (GA).

**Treatments and**		**Comet class**				**Scores**
**cells analyzed**	**Total1**	**0**	**1**	**2**	**3**	
**Peripheral blood (4h sample)**						
**Control**	7.00 ± 2.16	93.0 ± 2.16	5.33± 1.49	1.66± 0.94	0.00 ± 0.00	8.66 ± 2.98
**GA15 mg/kg**	8.83± 3.02	91.1± 3.02	8.50 ± 2.75	0.33± 0.74	0.00 ± 0.00	9.16± 3.43
**GA 30 mg/kg**	37.0± 5.88^c^	63.0 ± 5.88^c^	34.0± 6.11^c^	3.00± 1.82	0.00 ± 0.00	40.0± 6.21^c^
**GA60 mg/kg**	29.3 ± 4.06^c^	70.6± 4.06^c^	26.6 ± 3.34^c^	2.66± 1.49	0.00 ± 0.00	32.0± 5.13^c^
**Doxorubicin 80 mg/kg**	35.3 ± 5.76^c^	63.0 ± 4.00^c^	29.6± 4.92^c^	5.33 ± 2.89	0.33 ± 0.47	41.3 ± 8.05^c^
**Peripheral blood (24h sample)**						
**Control**	9.33 ± 1.97	90.6 ± 1.97	6.66 ± 1.49	2.66 ± 0.74	0.00 ± 0.00	12.0 ± 2.58
**GA15 mg/kg**	13.0 ± 3.26	87.0 ± 3.26	10.6 ± 2.80	2.33 ± 1.24	0.00 ± 0.00	15.3 ± 4.06
**GA 30 mg/kg**	17.8 ± 5.01^a^	82.1 ± 5.01^a^	16.1 ± 4.98^b^	1.66 ± 4.98	0.00 ± 0.00	19.5 ± 5.15
**GA60 mg/kg**	24.0 ± 3.60^c^	76.0 ± 3.60^c^	22.3 ± 2.98^c^	1.66 ± 1.24	0.00 ± 0.00	25.6 ± 4.49^a^
**Doxorubicin 80 mg/kg**	68.5 ± 5.79^c^	31.5 ± 5.79^c^	47.1 ± 6.14^c^	16.1 ± 4.29^c^	5.16 ± 3.07^c^	95.0 ± 14.0^c^
**Liver**						
**Control**	12.8 ± 3.02	87.1 ± 3.02	12.8 ± 3.02	0.00 ± 0.00	0.00 ± 0.00	12.8 ± 3.02
**GA15 mg/kg**	35.0 ± 5.62^c^	65.0 ± 5.62^c^	31.5 ± 4.64^c^	3.50 ± 1.25^b^	0.00 ± 0.00	38.5 ± 6.70^c^
**GA 30 mg/kg**	35.6 ± 4.88^c^	64.3 ± 4.88^c^	31.6 ± 3.49^c^	4.00 ± 1.63^c^	0.00 ± 0.00	39.6 ± 6.39^c^
**GA60 mg/kg**	45.8 ± 3.18^c^	54.1 ± 3.18^c^	40.8 ± 2.03^c^	5.00 ± 1.52^c^	0.00 ± 0.00	50.8 ± 4.56^c^
**Doxorubicin 80 mg/kg**	39.6 ± 4.71^c^	60.3 ± 4.71^c^	35.5 ± 4.07^c^	4.16 ± 1.46^c^	0.00 ± 0.00	43.8 ± 5.66^c^
**Bone marrow**						
**Control**	8.50 ± 2.75	91.5 ± 2.75	8.50 ± 2.75	0.00 ± 0.00	0.00 ± 0.00	8.50 ± 2.75
**GA 15 mg/kg**	30.8 ± 5.14^c^	69.1 ± 5.14^c^	26.8 ± 5.98^c^	4.00 ± 1.29^a^	0.00 ± 0.00	34.8 ± 4.52^c^
**GA 30 mg/kg**	17.6 ± 4.42^a^	82.3 ± 4.42^a^	14.0 ± 4.72	3.66 ± 1.37^a^	0.00 ± 0.00	21.3 ± 4.53^b^
**GA60 mg/kg**	19.6 ± 2.19^b^	80.3 ± 3.19^b^	14.1 ± 1.57	5.50 ± 2.21^c^	0.00 ± 0.00	25.1 ± 5.27^c^
**Doxorubicin 80 mg/kg**	36.0 ± 5.44^c^	64.0 ± 5.44^c^	28.6 ± 3.09^c^	7.00 ± 2.82^c^	0.33 ± 0.47	43.6 ± 8.65^c^
**Brain**						
**Control**	10.0 ± 1.29	90.0 ± 1.29	10.0 ± 1.29	0.00 ± 0.00	0.00 ± 0.00	10.0 ± 1.29
**GA 15 mg/kg**	37.0 ± 3.26^c^	63.0 ± 3.26^c^	34.3 ± 3.54^c^	2.66 ± 0.94^b^	0.00 ± 0.00	39.6 ± 3.24^c^
**GA 30 mg/kg**	39.1 ± 4.52^c^	60.8 ± 4.52^c^	36.8 ± 4.77^c^	2.33 ± 0.74^b^	0.00 ± 0.00	41.5 ± 4.38^c^
**GA60 mg/kg**	37.3 ± 2.56^c^	62.6 ± 2.56^c^	35.1 ± 2.91^c^	2.16 ± 1.46^a^	0.00 ± 0.00	39.5 ± 2.98^c^
**Doxorubicin 80 mg/kg**	40.5 ± 2.36^c^	59.5 ± 2.36^c^	35.5 ± 1.89^c^	5.00 ± 1.15^c^	0.00 ± 0.00	45.5 ± 3.20^c^
**Testicle**						
**Control**	8.33 ± 2.74	91.6 ± 2.74	8.33 ± 2.74	0.00 ± 0.00	0.00 ± 0.00	8.33 ± 2.74
**GA 15 mg/kg**	28.8 ± 3.89^c^	71.1 ± 3.89^c^	28.8 ± 3.89^c^	0.00 ± 0.00	0.00 ± 0.00	28.8 ± 3.89^c^
**GA 30 mg/kg**	30.8 ± 3.53^c^	69.1 ± 3.53^c^	30.8 ± 3.53^c^	0.00 ± 0.00	0.00 ± 0.00	30.8 ± 3.53^c^
**GA60 mg/kg**	33.8 ± 3.43^c^	66.1 ± 3.43^c^	33.8 ± 3.43^c^	0.00 ± 0.00	0.00 ± 0.00	33.8 ± 3.43^c^
**Doxorubicin 80 mg/kg**	45.3 ± 2.92^c^	54.6 ± 2.92^c^	40.6 ± 2.49^c^	4.66 ± 1.24^c^	0.00 ± 0.00	50.0 ± 3.74^c^

^a^ Significantly different from the negative control (P < 0.05); ^b^ Significantly different from the negative control (P < 0.01); ^c^ Significantly different from the negative control (P < 0.001) ^1^;Total number of damaged cells (class 1+2+3). Data presented as Mean ± Standard Deviation of the mean.

**Table 2:** Number of micronucleated polychromatic erythrocytes (MNPCE) observed in the bone marrow cells of male Swiss mice (M_1-6_) treated with Guttiferone A (GA), and respective controls.

**Treatments**				**Number of MNPCE per Animal**								**MNPCE**	**PCE/NCE**
												(Mean ± SD)	(Mean ± SD)
	M_1_				M_2_	M_3_	M_4_	M_5_	M_6_				
**Control**	3				1	2	3	2	2			2.16± 0.68	1.15± 0.04
**GA**	4				4	5	6	6	5			5.00± 0.81^a^	1.19± 0.06
**(15 mg/kg)**													
**GA**	6				6	5	4	5	5			5.16 ± 0.68^a^	1.25± 0.08
**(30 mg/kg)**													
**GA**	7				5	6	5	6	5			5.66 ± 0.74^a^	1.23± 0.03
**(60 mg/kg)**													
**Doxorubicin (DXR)**	10				11	11	12	10	11			10.83± 0.68^a^	1.18 ± 0.02
**(80 mg/kg)**													

^a^ Significantly different from the negative control (P< 0.001). Two thousand cells were analyzed per animal. SD = standard deviation from the mean.

 The frequency of micronucleated polychromatic erythrocytes (MNPCE) and PCE-NCE ratio in bone marrow cells of mice are presented in Table 2. The number of micronucleated cells increased significantly after treatment with 15, 30 and 60 mg/Kg b.w. of guttiferone A, demonstrating that the compound has effects on these mutagenic endpoints at the doses tested. Also, the MNPCE increase was not directly related to the tested doses. The administration of DXR resulted in a significant increase (P < 0.001) in micronucleated cells when compared with the negative control. The estimated ratio of PCE-NCE in 200 bone marrow erythrocytes/animal showed no statistically significant alterations in hematopoiesis as a result of guttiferone A or DXR treatment, indicating no cytotoxic effects (Table 2).

## Discussion

 Benzophenones are non-polar phenolic compounds, which show increased hydrophobicity as the number of attached prenyl functional groups increases. They are major intermediates in the biosynthetic pathway of xanthones, and are rarely reported to occur outside the Clusiaceae family [16]. Their potent biological properties have been the subject of several studies [7], and to our knowledge, there are few toxicological studies on natural polyisoprenylated benzophenones. Therefore, the aim of this study was to investigate the possible genotoxicity of benzophenone guttiferone A assessed in acute treatment, using the comet assay and micronucleus test in mice. Since the Guttiferone A isolated still is not used by humans, the treatment regimen and the administration method used in the present study was considered the most suitable for humans, and the tested doses were chosen on the basis of its gastroprotective effects evaluated in rodents [8].

 The purposes of toxicological genetic are to assess the mutagenicity of chemicals, physical, and biological agents in order to protect the human gene pool, and to identify potential carcinogens. The genotoxicity of environmental factors in mammalian cells can be determined in different ways: by identification of gene mutations, DNA breaks, DNA damage, DNA repair, chromosome aberrations, chromosome breakage and chromosome loss [17]. The *in vivo* alkaline version of the comet assay (single-cell gel electrophoresis) is increasingly being used in genotoxicity testing. Its advantages include its applicability to various tissues and/or cell types, detecting DNA damage such as strand breaks, alkali-labile sites, DNA-DNA and DNA-protein crosslinks [18]. In the present study, the results of our comet assay showed that the three tested doses of guttiferone A increased the DNA damage in leukocytes, liver, bone marrow, brain and testicle cells of mice, indicating a genotoxic effect of this compound.

 The other mutagenicity assay performed in this study was the micronucleus (MN) test. The assay in bone marrow erythrocytes is one of the most widely-used *in vivo* cytogenetic assays in the field of genetic toxicology. MN are expressed in dividing cells that either contain chromosome breaks lacking centromeres (acentric fragments) and/or whole chromosomes that are unable to travel to the spindle poles during mitosis (aneugenic effect) [19,20]. This other endpoint evaluated in the present study showed that guttiferone A produced chromosome breaks and/or aneugenic effects in bone marrow erythrocytes of mice.

 Similar results were obtained in the genetic toxicity evaluation of another benzophenone, garcinielliptone [21,22]. The authors observed that this compound produced nuclear fragmentation in breast cancer (MCF-7) cells. According to their findings, garcinielliptone generated reactive oxygen species, which caused the breakage of DNA and cell death. On the other hand, Almanza et al. [23] reported that the benzophenone acuminophenone A, and the xanthones formoxanthone C and macluraxanthone isolated from *Rheedia acuminate* showed no mutagenicity on several *Salmonella typhimurium* strains. Additionally, the authors also observed that these compounds promoted a strong reduction of mutagenic effect induced by hydrogen peroxide.

 Regarding the genetic toxicity assessment of plant extracts belonging to *Garcinia* or *Rheedia* genus, our literature review found only one study, developed by our own research group. The *in vivo* evaluation of a single oral administration of *Garcinia achachairu* seed extract to mice showed that even high doses of the extract did not cause genotoxicity and clastogenicity in different cells of mice, by the comet and MN assays [6].

 Considering that DNA-protective compounds that interact directly with mutagens are classified as desmutagens [24], we can hypothesize that possible chemical interaction processes between the components of this extract may be exerting some desmutagenic effect on guttiferone A, which explains the differences in the genotoxicity results observed between the crude extract and its isolated compound. This finding needs to be investigated in further studies.

 In conclusion, the genotoxic assessment performed in the present study demonstrated, for the first time that, that a single oral administration of guttiferone A obtained from the seeds of *Garcinia achachairu* produces genotoxic effects in leukocytes, liver, bone marrow, brain and testicle cells and clastogenic/aneugenic effects in bone marrow erythrocytes of mice. Therefore, despite the fact that guttiferone A represents a promising medicinal natural compound with analgesic and gastroprotective profiles, based on its genetic toxicity observed in our study, we recommend caution in the acute or chronic use of this compound. 
